# What Is Speciation?

**DOI:** 10.1371/journal.pgen.1005860

**Published:** 2016-03-31

**Authors:** B. Jesse Shapiro, Jean-Baptiste Leducq, James Mallet

**Affiliations:** 1 Département de sciences biologiques, Université de Montréal, Montréal, Quebec, Canada; 2 Department of Organismic and Evolutionary Biology, Harvard University, Cambridge, Massachusetts, United States of America; Université Paris Descartes, INSERM U1001, FRANCE

## Abstract

Concepts and definitions of species have been debated by generations of biologists and remain controversial. Microbes pose a particular challenge because of their genetic diversity, asexual reproduction, and often promiscuous horizontal gene transfer (HGT). However, microbes also present an opportunity to study and understand speciation because of their rapid evolution, both in nature and in the lab, and small, easily sequenced genomes. Here, we review how microbial population genomics has enabled us to catch speciation “in the act” and how the results have challenged and enriched our concepts of species, with implications for all domains of life. We describe how recombination (including HGT and introgression) has shaped the genomes of nascent microbial, animal, and plant species and argue for a prominent role of natural selection in initiating and maintaining speciation. We ask how universal is the process of speciation across the tree of life, and what lessons can be drawn from microbes? Comparative genomics showing the extent of HGT in natural populations certainly jeopardizes the relevance of vertical descent (i.e., the species tree) in speciation. Nevertheless, we conclude that species do indeed exist as clusters of genetic and ecological similarity and that speciation is driven primarily by natural selection, regardless of the balance between horizontal and vertical descent.

## How Many Species and How Much Speciation?

Conservatively assuming there are ~10^7^ different species on Earth, not counting most bacteria and archaea [1], and a single origin of life ~4x10^9^ years ago, this gives an average diversification rate of 0.0025, or one new species every 400 years. This estimate is very rough and does not account for extinction events or “bursts” of speciation, and it is likely a severe underestimate because microbes are undercounted. More impressive than the number of species is the number of intermediate forms—Darwin’s “doubtful cases” [2]—suggesting that speciation is a continuous process that happens all the time [3]. This apparent fluidity has led us and others to propose that most organisms can probably be placed somewhere along a “spectrum” of speciation [4,5]. Of course, speciation may not happen at all, or at least not go to completion. Here, we are less concerned with the number and exact definition of species and more with why speciation happens (or not) and the nature of the speciation process.

## A Brief History of Species Thinking

Here, we consider species in the vernacular sense, as clusters of individuals that show ecological and genetic similarities. We tend to know them when we see them—although microbial species are more difficult to “see” than those of multicellular eukaryotes (referred to here as macrobes). Given that species evolve from common ancestors (an evolutionary and phylogenetic species definition, e.g., [6]), the big question is not so much what species are, but what evolutionary forces make them (and keep them) distinct?

Darwin emphasized the role of natural selection and competition in shaping species and keeping them in separate ecological niches. Dobzhansky [7] and Mayr [8] emphasized the importance of reproductive isolation in maintaining the genetic distinctness of species; this “biological species concept” (BSC) based on sexual isolation does not easily apply to asexually reproducing organisms, including most bacteria and archaea (Box 1). Simpson [9] suggested more generally that distinct species must have separate evolution, and Van Valen [10] argued that this separateness is mainly due to ecological distinctness, not to reproductive isolation. Throughout this article–while acknowledging that reproductive isolation also involves selection (e.g., negative selection against Dobzhansky–Muller incompatibilities [11]–we use the term “natural selection” or simply “selection” to mean differential selection in different ecological niches. We also refer generically to "gene flow" or "genetic exchange," whether it involves the exchange of different alleles of homologous genes (similar to meiotic sex [12]) or the acquisition of brand new genes by nonhomologous recombination.

**Box 1. Glossary**

**Allopatric:** a set of sampled isolates or genomes from different geographic areas, where barriers to migration and gene flow are significant.

**Biological species concept (BSC):** a species concept based on restricted gene flow, in which genes are exchanged by recombination within but not between species. In sexual species, this is equivalent to sexual or reproductive isolation. In asexually reproducing (clonal) species, a version of this concept could apply when there is more HGT within than between species.

**Clonal frame**: the portion of the genome transmitted by vertical (clonal) evolution, unimpacted by HGT. Mutations in the clonal frame should all fall parsimoniously on a single phylogenetic tree. The concept of clonal frame is related to, but not identical to, the concept of core genome, which is the portion of the genome that is present (or in practice, that can be aligned) in all of a given set of sequenced isolates or metagenomes. The core genome is not necessarily vertically inherited and is therefore not necessarily part of the clonal frame.

**CRISPR:** Clustered, regularly interspaced short palindromic repeats in the genome, which, along with associated protein-coding genes, confer many bacteria and archaea with a type of adaptive immunity to mobile genetic elements.

**Darwinian Threshold:** the transition from mostly horizontal to mostly vertical transmission of genetic material, allowing the possibility of a branching tree structure relating species.

**Exaptation:** the process in which DNA or genes originally selected for one function (or originally selectively neutral) are selected for a new and different function.

**Gene flow**: exchange of genes by homologous or nonhomologous recombination

**Gene-specific selective sweep:** the process in which a selected gene or allele spreads in a population by recombination faster than by clonal expansion. The result is that the selected variant is present in more than a single clonal background, and diversity is not purged genome-wide when the selected gene reaches fixation.

**Genetic drift:** the tendency for units (mutations, genes, or individuals) to change in frequency because of random sampling in a population of finite size.

**Genome-wide selective sweep:** the process in which a selected gene or allele spreads in a population by clonal expansion of the genome that first acquired it. The result is that diversity is purged genome-wide, and the selected variant is linked in the same clonal frame as the rest of the genome.

**Hologenome:** the total set of genomes contained in a host and its symbionts (e.g., an animal's nuclear and mitochondrial genome, plus the genomes of its symbiotic microbiota).

**Horizontal gene transfer (HGT):** the incorporation of foreign DNA into a genome. Incorporation can be mediated by either homologous recombination or nonhomologous recombination of DNA that enters a cell via transformation, transduction, conjugation, or other mechanisms. In bacteria and archaea, all gene transfer is horizontal (i.e., always unidirectional from donor to recipient, rather than reciprocal). Horizontal transmission occurs within a generation, as opposed to vertical transmission of DNA from one generation to the next.

**Homologous recombination:** a mechanism of DNA integration requiring at least short tracts of identity between the genome and the foreign DNA, mediated by RecA and mismatch-repair machinery. The integrated DNA can result in single-nucleotide changes and, in some cases, addition or loss of hundreds to thousands of base pairs.

**Hybridization:** in sexual organisms, the process in which two individuals from distinct (but typically closely related) populations or species form viable progeny (hybrids) harboring a combination of both parental genomes.

**Introgression (or introgressive hybridization):** in sexual organisms, the process in which genes or portions of the genome are transferred from one population (or one species) to another by hybridization, followed by successive backcrosses with parental genomes.

**Macrobe**: a multicellular eukaryote.

**Microbe:** a microscopic single-celled bacterium, archaean, or eukaryote.

**Mobile genetic element:** a piece of DNA that is frequently transferred horizontally, either within or between genomes, and often encodes its own replication and transfer (e.g., plasmids, phages, transposons, integrative conjugative elements).

**Natural selection:** differential survival and reproduction of units (mutation, genes, or individuals) from one generation to the next.

**Negative frequency-dependent selection (NFDS)**: a type of natural selection that favors rare phenotypes in a population.

**Niche:** a specific set of ecological parameters (environments, resources, physical and chemical characteristics, biotic interactions, etc.) to which an organism is adapted. This does not necessarily imply (but does not exclude) physical separation between niches.

**Nonhomologous recombination:** integration of DNA with no homologous allele already present in the genome, often mediated by phage and integrative elements. This results in the acquisition of entirely new genes.

**Population:** a group of individuals sharing genetic and ecological similarity and coexisting in a sympatric setting.

**Species:** a group of genetically and ecologically similar individuals that may be named with a Linnean binomial to aid communication. Species are recognizable as distinct clusters, based on genetic similarity across the genome and differences from other species. In most cases, distinct genetic clusters imply distinct ecology between clusters, otherwise clusters will not form or persist. These genetic clusters can be large (encompassing a great deal of genetic diversity) or small, and may contain ecological diversity that may eventually drive speciation (separation of one cluster into two) or may not (gene ecology).

**Sympatric:** a set of sampled isolates or genomes from the same geographic area, where barriers to migration and gene flow are low or nonexistent.

**Taxon:** a group of biological entities (species, genera, class, etc.) deriving from the same ancestor, defined by shared characteristics inherited from this ancestor.

Van Valen went on to speculate, “It may well be that *Quercus macrocarpa* in Quebec exchanges many more genes with local *Q*. *bicolor* than it does with *Q*. *macrocarpa* in Texas.” His idea—that gene exchanges (whether mediated by homologous or nonhomologous recombination) occur more frequently according to ecology and local geography than according to species boundaries—has been supported in genomic surveys of natural microbial populations. For example, we could simply replace some nouns in Van Valen’s quote to produce the following statement: *Vibrio cholerae* in the United States exchanges more genes with local *V*. *metecus* (a sister species) than it does with *V*. *cholerae* in Bangladesh [13]. Similar examples are found in animals such as *Heliconius* butterflies [14]. However, only a certain subset of genes is shared along geographic and/or ecological lines, while the rest of the genome evolves according to established (named) species boundaries. *V*. *cholerae* and *V*. *metecus* are therefore “good” species, recognizable as distinct genetic and ecological clusters despite exchanging genes for local adaptation. As we will see below, earlier stages of speciation are often characterized by the opposite genomic signature: only a subset of genes are diverged between species while the rest of the genome is freely recombined across species.

Van Valen also coined the term “multispecies”: a set of broadly sympatric species that exchange genes in nature. This term should resonate with microbial ecologists familiar with the famous trope, “Everything is everywhere [i.e., sympatric], but the environment selects” [15]. As the potential for global dispersal and widespread horizontal gene transfer (HGT) becomes increasingly apparent, it is not implausible to consider all bacteria, or even all life on Earth, as a sort of multispecies. Van Valen did not go quite so far, but did suggest that there could be taxa without species and that the family Enterobacteriaceae, for example, might constitute one such multispecies unit. We disagree that there are taxa without species. However, if a pair of putative species is discovered to form a single genetic cluster (for example, if unable to be distinguished in an assignment test such as BAPS [16] or STRUCTURE [17]), we should conclude that there is one species, rather than no species or multispecies. Our perspective implies that some species may contain much more genetic diversity than others and that a simple operational cutoff of percent DNA identity would not be appropriate for species delimitation.

Finally, Van Valen observed that “multispecies seem to occur less commonly among metazoans than elsewhere” and suggested that this could be due to increased complexity and precise mating systems in metazoa. This concept of speciation as a byproduct of biological complexity rather than ecology was explored and elaborated in Woese’s idea of “Darwinian Threshold” [18], referring to the transition from a precellular soup with rampant HGT to a mostly tree-like pattern of distinct species that undergo distinguishable speciation events. According to Woese, once the complex machinery of replication and protein translation had evolved, it became “locked in place” by coadaptation, and its individual components could not be easily horizontally transferred from cell to cell because they would be incompatible with divergent recipient cell machinery. Consistent with the logic of the complexity hypothesis [19], HGT is more common among genes that function at the periphery rather than the highly interconnected core of bacterial metabolic networks [19,20]. However, the cumulative impact of HGT on the tree of life is much greater than imagined by Woese. The tree of life has been criticized as “the tree of one percent” [21] because only about 1% of genes support this tree [22]. While HGT has not obscured all traces of vertical descent across the tree of life [23,24], much of any organism’s genome may not have crossed the Darwinian Threshold, and may never do so.

In contrast to Van Valen’s selection-driven ecological speciation (and Cohan’s subsequent ecotype models [25]), neutral speciation involves only genetic drift. A typical neutral scenario would be a population that becomes geographically separated, allowing the two sub-populations to diverge genetically, such that they become reproductively incompatible if and when they meet again. Speciation affected by neutral processes is expected to be more common in macrobes because of populations with strong biogeography (limited dispersal) and smaller effective population sizes that favor drift over natural selection. Lynch and Conery [26] even suggest that drift was the major factor leading to evolutionary diversification in macrobes, with the neutral accumulation of noncoding DNA leading to increasing genome expansion, allowing complex gene regulation and cell specialization and in turn leading to exaptation of ecological novelties.

According to the "everything everywhere" dogma, most microbes form populations large enough to accumulate mutations that could be beneficial in a broad range of environments and to migrate so efficiently that few genetic incompatibilities have a chance to fix via drift within populations. Speciation in the microbial world is therefore expected to involve little drift and geographical separation. However, drift plays an important role in the evolution of microbial symbionts and pathogens that undergo population bottlenecks during transmission from host to host [27]. Drift may therefore play a dominant role in the evolution of endosymbionts such as *Buchnera* [28], but this does not necessarily exclude a role of natural selection in their speciation. Some microbes also have strongly constrained geographic distributions. For example, thermophilic archaea diverge genetically with geographic separation [29,30]. Some yeasts also experience limited migration across continents [31,32] and population size fluctuations [33,34], both of which may contribute to the emergence of species. However, strong selection, for instance driven by domestication [31] or local climatic adaptation [35], can either reinforce or mitigate speciation in yeast. Hence, as in macrobes, speciation in microbes will be driven by a balance between drift and selection, with macrobes likely experiencing more drift because of smaller population sizes and limited dispersal.

**Fig 1:**
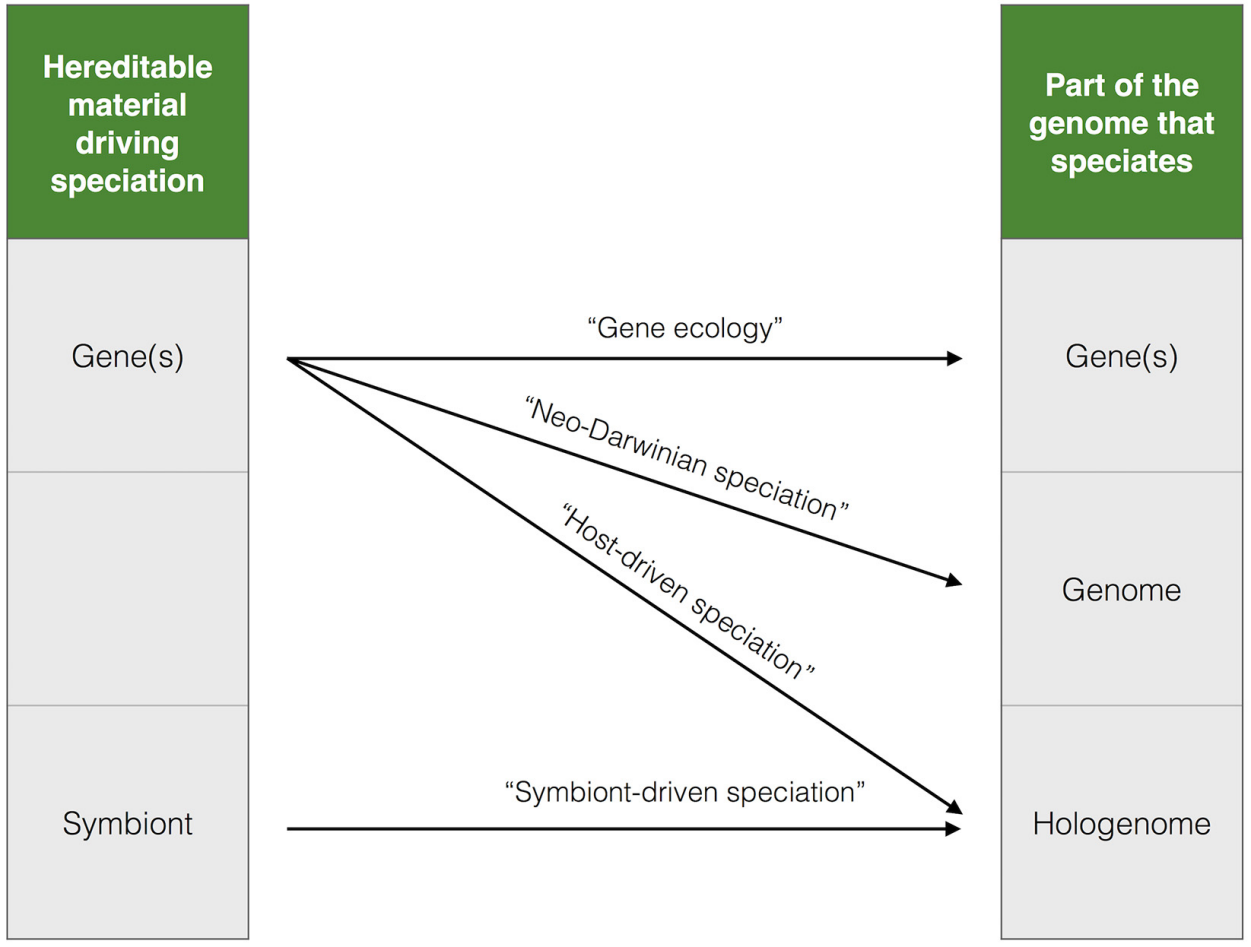
Units of species and speciation. The Neo-Darwinian view of the Modern Synthesis is that "speciation genes" are the units driving speciation across the genome. Alternatively, if gene sets (including consortia of genes like plasmids or other mobile genetic elements) are sufficiently decoupled from their host genomes, this will lead to "gene ecology," in which gene sets, not species, determine reproductive isolation and/or adapt to ecological niches. Speciation could also be maintained (or potentially driven) by microbial symbionts or by host genes that select for particular symbionts, resulting in hologenome species. All of these speciation mechanisms can potentially be driven by selection or drift, and the list of units and mechanisms (arrows) is not exhaustive.

More broadly, the species problem can be viewed as a specific instance of the “levels of selection” problem [36,37]: how do natural selection and drift act on units at different levels of organization—ranging from genes, to protein complexes, to cells, to populations, to communities—to yield cooperation and cohesiveness within units but boundaries between units? It also raises the question, what are species made of? The Neo-Darwinian perspective (resulting from the Modern Synthesis of Darwinism and Mendelian genetics [38,39]) is that species differ genetically across their whole genomes, and speciation is caused by "speciation genes"—some combination of genes that cause reproductive isolation and/or adaptation to different ecological niches (Fig 1). Traditionally, populations of organisms have been viewed as the units undergoing speciation, with whole-genome isolation developing between them. However, the lack of support for a cleanly branching organismal phylogeny has suggested to some that we should think of speciation as applying only to parts of the genome, not the whole genome—the “genic view” of speciation [4]. In essence, different parts of the genome may speciate at different rates or not at all [40], such that variable sets of genes are the elements that truly speciate (Fig 1). In the genic view, speciation still occurs but is driven by natural selection on genes, while reproductive isolation can remain incomplete. Taken to an extreme, this becomes “gene ecology” [25,41], and speciation does not occur. Rather, a set of genes or alleles inhabits the ecological niches to which they are best adapted without driving isolation of the rest of the genome. For example, vancomycin resistance genes might inhabit the hospital niche, and otherwise identical strains of *Staphylococcus aureus* may differ only in the presence or absence of these genes [42]. We might not classify these strains as separate species, but with time, their ecological differences could be followed by genetic differentiation and speciation. Symbiotic microbes might also maintain species boundaries, leading to the concept of holobionts: species that are made of multiple genomes, including host and symbionts [43–45]. Holobiont concepts are still in their infancy [46], and the extent of their contribution to speciation will surely become clearer in the coming years. Thus, the populations we call species can vary widely in what fractions of their genomes and hologenomes are isolated and how they emerge and remain isolated.

## Are Eukaryotes Fuzzy Like Bacteria?

Since Dobzhansky and Mayr, the prevailing dogma has been that bacteria are “messy” because they don't easily fit the BSC. Recent findings are challenging this dogma, showing that while species are indeed messy in bacteria, they can be almost as messy in eukaryotes [12]. In other words, bacteria may fit the BSC better than we had thought [5,47,48] and eukaryotes may fit it worse. Eukaryotic genomes are impacted by HGT from viruses, bacteria, and even other eukaryotes [49,50]. Mobile genetic elements make up about two-thirds of the human genome, and their origins are often due to HGT [51–53]. HGT in eukaryotes, even if rare, can be important in the gain of new functions and, potentially, in speciation. Even without invoking interdomain HGT, gene flow by sexual hybridization across eukaryotic species boundaries (introgression) can be strong enough to obscure species branching events in large regions of the genome. In some cases, introgressive gene flow can bring new traits to a species, potentially giving rise to new varieties or even new species [34]. For example, HGT among close (introgression) or distant species of fungi, and even between fungi and bacteria, together with chromosomal rearrangements, have substantially shuffled fungal genomes and contributed to the emergence of new phytopathogenic [54,55] and brewing species [33,56]. In other cases, introgression (usually between closely related species pairs) has the potential to merge two species into one (e.g., [57]). It can be difficult to distinguish whether introgression is leading to genome-wide species convergence or simply the exchange of a few loci in the genome. For example, two species of *Campylobacter* were proposed to be converging [58], but the convergence may be at a very early stage or may simply involve the exchange of a few environmentally adaptive genes [59].

Although species boundaries are generally considered less fuzzy in macrobes, gene transfers by introgression among related species were revealed by fuzzy phylogenetic signals in genomic regions containing genes involved in mimicry in *Heliconius* [60,61] and in altitude adaptation in humans [62]. Hybridization and introgression may occur among non-sister species as well as well as between sister species, especially during rapid adaptive radiations. For example, in *Heliconius*, the "*melpomene*-silvaniform" clade consists of around 15 species. Most of these are "good" species that co-occur over large sympatric regions and are somewhat interfertile with other members of the clade. However, hybrids and backcrosses across the entire group occur in the wild and in captivity, suggesting the possibility that a slow trickle of introgression may be constantly occurring among both close and distant relatives [63]. In mosquito species, only a small fraction of the genome, mainly on the X chromosome, has not crossed species boundaries [64]. Yet, these mosquito species still form clear and distinct genetic clusters, thus fitting the criteria of “fuzzy species,” as originally proposed for macrobes [65] and microbes [66]. This is not to say that all eukaryotes form fuzzy species, nor all bacteria—rather, fuzzy species may emerge across the entire tree of life, given the right regime of recombination (HGT or gene flow).

## The Islands Debate

**Fig 2:**
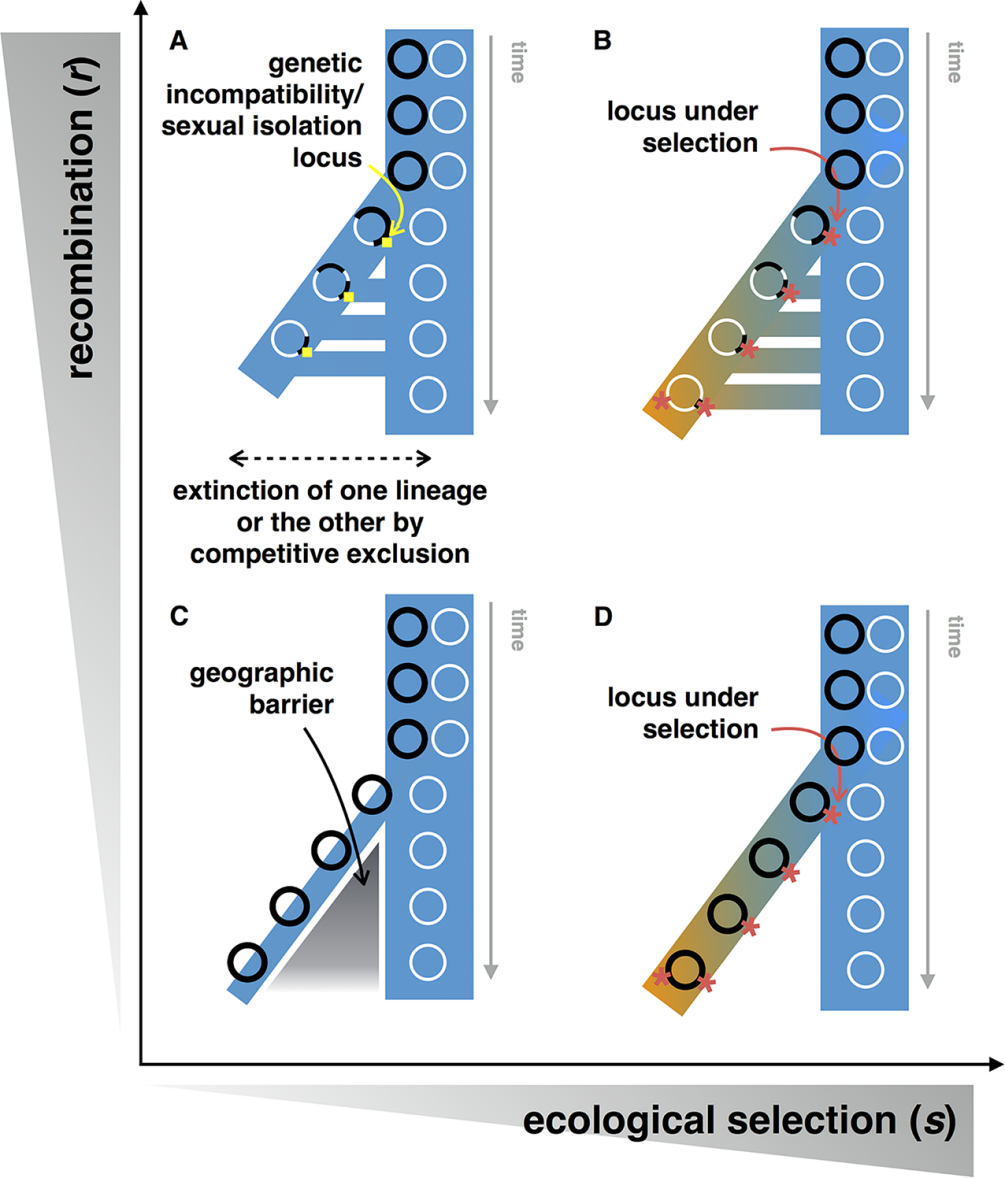
Models of speciation under different regimes of selection and recombination. In all models, a single population of chromosomes (circles) splits into two nascent species, distinguishable by sets of genetic differences. At each time point, the most frequent multilocus genotype is shown, but other chromosomes could be segregating in the population at lower frequencies. Different haplotypes (or clonal frames) are shown as black or white circles. The ancestral niche is shown in blue and a new niche in orange. Gene flow (recombination) between species is indicated by horizontal connections between branches. (**A**) In the simplest model of speciation with gene flow, a single mutation controlling sexual isolation (but not under selection) is the only divergent locus (yellow square), with other loci experiencing gene flow between incipient species. (**B**) Selection during speciation can produce a pattern of genetic diversity across the genome very similar to (A), but species are expected to be longer-lived. Mutations under selection at early and later stages of speciation are shown as orange stars. (**C**) Allopatric speciation with a population bottleneck and neutral divergence of species. As in (A), competitive exclusion should lead to the extinction of one species if they come back into contact. (**D**) Without gene flow, the mutation under selection between species (orange star) will purge diversity genome-wide as it sweeps through one population, resulting in genome-wide divergence from the other population.

Most of the initial research and theory on speciation focused on plant and animal populations, with one of the major debates centered on the relative importance of sympatric and allopatric speciation. Under the BSC, allopatry (physical separation, e.g., by islands or mountain ranges) provides a simple mechanism of reproductive isolation (Fig 2). Sympatric speciation, in the absence of barriers to gene flow, was initially thought to be rare, but more and more examples are being found in eukaryotes, either involving hybrid speciation [67,68] or not [69–72].

Genomic comparisons of putative sympatric species pairs have revealed so-called genomic “islands of speciation,” parts of the genome that are highly divergent between species, while the rest of the genome is undifferentiated. Islands are thought to contain genes driving reproductive isolation [73]. As a result, islands are resistant to gene flow during speciation, while the rest of the genome is more likely to acquire genes across incipient species boundaries. The “speciation-with-gene-flow” model has been criticized as a potential artefact of a measure of genetic differentiation used to detect islands, and islands might appear because of lowered levels of polymorphism rather than as a result of any gene flow between species [74,75]. In the simplest model with gene flow but without selection, incipient species inhabit the same ecological niche (Fig 2A). As a result of competitive exclusion, one species will eventually go extinct [76] and speciation will fail. For speciation to succeed in the longer term, there should be at least some ecological differentiation between species, and islands should contain genes under divergent natural selection (Fig 2B).

## Islands in Bacteria

Genomic regions akin to islands of speciation have also been described in natural microbial populations (reviewed in detail in [5]). Briefly, both *Sulfolobus* archaea [48] and *Vibrio* bacteria [47] have parts of their genomes that are strongly differentiated along ecological lines, whereas the rest of the genome remains undifferentiated and freely recombined between ecologically distinct strains. However, both *Vibrio* and *Sulfolobus* show a recent and increasing tendency for gene flow within rather than between ecological populations—a pattern reminiscent of the BSC. In *Sulfolobus*, the differentiated regions (defined as having high relative divergence) encompass approximately one-third of the genome, making them more analogous to continents than islands. In *Vibrio*, the islands occupy only about one percent of the genome and were defined as regions of high absolute divergence between ecological populations. The *Vibrio* islands were likely acquired from HGT from another *Vibrio* species, analogous to speciation by introgression in macrobes [60,77].

At first glance, these observations support some flavor of the speciation-with-gene-flow model for *Vibrio* because of its small islands of high absolute divergence (Fig 2A). For *Sulfolobus*, with its large continents of high relative divergence, distinguishing among models is more difficult. The two *Sulfolobus* populations could potentially have diverged in allopatry (e.g., in separate hotsprings) before encountering each other and exchanging genes in the hotspring from which they were sampled (Fig 2C). However, the *Sulfolobus* populations had different growth dynamics in the lab, suggesting ecological differences and a role for natural selection in keeping them separate [48].

In the BSC, speciation is initiated by boundaries to gene flow, perhaps followed by divergent natural selection. In the genic view, speciation is initiated by natural selection on genes, and reduced gene flow is a by-product, not a driver [4]. In the *Vibrio* populations, the island genes do not directly encode gene flow boundaries but likely provide adaptations to different ecological niches [78], resulting in divergent natural selection. Therefore, ecological speciation [79] might apply: islands arise because of divergent natural selection during speciation (Fig 2B). In this model, gene flow boundaries emerge later—as a consequence of less frequent encounters between strains with different ecological niches—or not at all. If complete boundaries to gene flow take some time to emerge, we can think of gene sets rather than whole genomes as the units that inhabit ecological niches. If gene flow boundaries never emerge, speciation does not occur (i.e., we are left with one species, not two) and this corresponds to the gene ecology model.

## Gene Sweeps Versus Genome-Wide Sweeps

With relatively high rates of recombination (*r*), individual genes will “sweep” to fixation in ecological niches to which they are adapted, and this will occur without affecting genetic diversity elsewhere in the genome. When rates of recombination are relatively low compared to selective coefficients (*s*) within niches, entire genomes will sweep to fixation before they can be shuffled by recombination. The *s* >> *r* regime is well described in the Stable Ecotype Model [25], which predicts that most of the genome will follow a single “clonal frame” phylogeny (Fig 2D).

Gene-specific selective sweeps were initially thought to be unlikely because recombination rates in microbes are estimated to be low (*r* < 10^−6^ per locus per generation) relative to selection (*s* > 10^−3^) [25]. However, recent modeling work [80] has shown that gene sweeps can occur when *r* is either very high or—counter-intuitively—when *r* is very low, but only in the presence of negative frequency-dependent selection (on other loci in the genome, in addition to positive selection on an ecologically adaptive locus). Such frequency-dependent selection, liable to be common in nature, might be imposed by viral (phage) predation of bacteria, providing a selective advantage to rare alleles of phage receptor genes, for example [81,82].

Additional sampling and sequencing from natural populations will be required to assess the prevalence of gene sweeps. One recent study described a “quasi-sexual” cyanobacterial population, in which virtually every gene in the genome was unlinked by recombination, with each sampled genome being a random combination of alleles [83]. Some of these alleles showed evidence of natural selection, suggesting the action of gene sweeps within a single cohesive population (i.e., gene ecology not leading to speciation).

## Open Questions

These recent models [80] and empirical work [83] have made some headway in resolving the paradox of gene sweeps but also raise new questions. How common are gene-sweeps relative to the genome-wide sweeps predicted by the Stable Ecotype Model? On what time scales do sweeps occur, and how does this affect speciation rates?

More generally, can all life on Earth, including microbes and macrobes, be viewed on the same universal speciation spectrum? Early stages on the spectrum involve natural selection and drift within a single population, in which diversity arises from mutation and/or recombination of both small [84] and large [85] pieces of both homologous and nonhomologous DNA. This genetic diversity can be neutral or selfish, consisting of mobile elements that could potentially (but not necessarily) be exapted for species-level adaptation. Later stages of speciation involve divergent natural selection and barriers to gene flow. The extent to which these barriers are ecological, behavioral, physical, or genetic remains an open research question. Evidence from comparative genomics has shown that purely genetic barriers such as CRISPR may provide effective barriers over short (within-species) time scales [86] but not over longer evolutionary time scales [87]. Therefore, gene flow barriers will always be leaky—in both microbes and macrobes.

Here, we have argued that selection, except in special cases of sustained allopatry, is almost certainly required for the long-term success of speciation. More examples will be needed to test its generality, but our model is as follows. Selection drives speciation and is followed by genome-wide divergence, due to reduced gene flow (in recombining populations) or mutational divergence (in clonal populations). If genome-wide divergence does not follow, speciation does not occur (or is stalled at a very early stage) and we are left with gene ecology. Just how much selection (on how many genes) and how much divergence across the genome is needed for speciation is an open question. Another important question is, for a given sample of organisms, what fraction of the genome is shaped by selection or drift within the individual, the species, or the multispecies [37]? In asking (and eventually answering) this question, we begin to appreciate that not only does speciation occur along a spectrum, but species can be placed within a spectrum of biological diversity, from the molecule to the biosphere.
